# Comparing the effectiveness of three scoring systems in predicting adult patient outcomes in the emergency department

**DOI:** 10.1097/MD.0000000000014289

**Published:** 2019-02-01

**Authors:** Xiaojun Wei, Haoli Ma, Ruining Liu, Yan Zhao

**Affiliations:** a Emergency Center, Zhongnan Hospital of Wuhan University; b Department of Biological Repositories, Zhongnan Hospital of Wuhan University, Wuhan, China.

**Keywords:** emergency department, outcomes, predictor, score system

## Abstract

This study aimed to evaluate the performance of the rapid acute physiology score (RAPS), the rapid emergency medicine score (REMS), and the modified early warning score (MEWS) in predicting the outcomes of adult patients presenting to the emergency department (ED).

A retrospective review was undertaken between February 2014 and February 2018 in an adult ED of a 3300-bed university hospital. The RAPS, REMS, and MEWS were calculated to assess their capability to predict hospital admission, length of hospital stay, and in-hospital mortality, using area under receiver operating characteristic analysis. Multivariate analysis was used to identify variables that were independent predictors of the outcomes.

We included 39,977 patients who had presented to the ED during 48 consecutive months, of whom 4857 were admitted and 213 died in hospital. The predictabilities of REMS, RAPS, and MEWS for hospital admission were 0.76, 0.59, and 0.55, respectively; the predictability of REMS, RAPS, and MEWS for hospital mortality were 0.88, 0.72, and 0.73, respectively; and the predictability of REMS, RAPS, and MEWS for length of hospital stay were 0.76, 0.67, and 0.65, respectively. Multivariate analysis showed that the Glasgow coma scale (GCS) (odds ratio (OR), 1.61; *P* < .001), age (OR, 1.50; *P* < .001), and MAP (OR, 1.27; *P* < .001) were independent predictors for hospital admission; GCS (OR, 2.92; *P* < .001), respiratory rate (RR) (OR, 2.69; *P* < .001), peripheral oxygen saturation (OR, 2.67; *P* < .001), MAP (OR, 2.11; *P* < .001), age (OR, 1.75; *P* < .001), and pulse rate (PR) (OR, 1.73; *P* < .001) were independent predictors for in-hospital mortality; and RR (OR, 1.41; *P* < .001), temperature (OR, 1.05; *P* = .01), and PR (OR, 0.96; *P* = .04) were independent predictors for length of hospital stay.

Our study evaluated and confirmed the REMS as a powerful predictor of ED adult patient outcomes, including hospital admission, length of hospital stay, and in-hospital mortality compared to RAPS and MEWS.

## 1 Introduction

The emergency department (ED) plays an important role in the management of ED patients with acute, complex, and changeable conditions.^[1]^ An accurate assessment of outcomes is imperative because it can promote early appropriate interventions and improve the outcomes of ED patients.^[2]^ There are certain disease states and conditions for which evaluation methods are appropriate and effective, such as ST-elevation myocardial infarction (STEMI), sepsis, and acute stroke. Moreover, several physiologic scoring systems have been demonstrated to be appropriate predictors of mortality for patients admitted to the ED. Among these scoring systems, the rapid acute physiology score (RAPS), the rapid emergency medicine score (REMS), and the modified early warning score (MEWS) are most commonly used for ED patients. These systems share the same characteristics and are comprised of simple physiological parameters that can be obtained rapidly, thus allowing quick clinical assessment for a critically ill patient who requires an urgent intervention.^[3]^ The MEWS system appears to be useful in predicting the outcomes of patients in a prehospital setting, but further studies are needed to determine its usefulness in the ED. The RAPS system was derived from the acute physiology and chronic health evaluation scoring system (APACHE-II), which has been verified as a reliable tool to determine the prognosis of patients. The REMS adds peripheral oxygen saturation and age to the RAPS.^[4]^ While all of these scoring systems assist emergency physicians, all the scores have significant shortcomings, such as difficulty in clinical application, regional variations, or in the statistical methods applied.^[5]^ Variables also have inherent flaws; they are associated with prognosis but cannot independently predict outcomes reliably. The external validity of these variables may also be questionable because their application in the ED setting has not been rigorous. Therefore, it is important to examine the performance of RAPS, REMS, and MEWS in predicting hospital admission, length of hospital stay, and in-hospital mortality for adult patients in the ED. Hence, this study was conducted for this purpose.

## 2 Methods

### 2.1 Study design

This was a retrospective review study of adult patients in the ED of Zhongnan Hospital (3300 beds with approximately 4400 ED visits monthly, Wuhan, China) between February 26, 2014 and February 28, 2018. Relevant data were extracted from a structured electronic medical records system and the data we collected included name, age, triage, sex, respiratory rate, pulse rate, heart rate, temperature, pulse oxygen saturation, blood pressure, and GCS details, which had been stored for ED patients in our institution. There were varying rates of missing data among these variables.

### 2.2 Settings and patients

We excluded the following patient groups:

1. patients clinically coded as having trauma (road traffic accidents, falls, assaults, or burns), mental illness, chest pain, or cardiac arrest, as there were relevant diagnoses and therapeutic centers within our hospital;
2. patients with no recorded clinical code;
3. patients who were dead at the emergency scene or dead on arrival at hospital;
4. those whose outcomes had not been recorded.

An adult patient was defined as a patient >18 years old. In-hospital mortality was defined as death occurring between the date of arrival and the date of hospital discharge. Length of hospital stay (LOS) was defined as the time between the date of arrival to hospital and the date of hospital discharge.

### 2.3 Measurement of variables

Variables were converted from numeric variables into categorical variables, and then the variables of RAPS, MEWS, and REMS were summed to calculate the overall scores accordingly. The RAPS has four variables, and the maximum score is 16; the REMS has six variables, and the maximum score is 26, and; the MEWS has 5 variables, and the maximum score is 14 (data not shown).^[1,3]^

### 2.4 Statistical analysis

Statistical analysis was performed using SPSS 24.0 (SPSS Inc., Chicago, IL) software. In all the analyses, we used the variables as they had been categorized, rather than raw data. Multivariate analysis was used to identify which variables were independent predictors of the outcomes. The area under receiver operating characteristic (AUROC) analysis was used to compare the predictive power among the scoring systems. In addition, sensitivity, specificity, and accuracy rates were calculated based on the optimal cut-off value. Odds ratios (ORs) with 95% confidence intervals (CIs) were calculated. *A P* value < .05 was considered statistically significant.

## 3 Results

### 3.1 Patient characteristics

A total of 39,977 patients meeting the study criteria and having presented to the ED for 48 consecutive months were included in the study, and we calculated the statistical associations between the three scoring systems and the major adverse outcomes, of which 21.8% involved respiratory disease, 17.9% involved digestive disease, 14.8% involved circulatory disease, and 1.6% involved poisoning. A total of 52.1% of the patients were female, and 4857 patients were admitted to hospital, 269 patients were admitted to the intensive care unit (ICU), and 213 patients died in the hospital. The mean age of the study patients was 44.5 ± 18.3 years, the mean temperature was 36.7 ± 0.9°C, the mean pulse rate was 84.6 ± 16.6 beats/min, the mean respiratory rate was 18.5 ± 2.7 breaths/min, the mean arterial pressure was 91.0 ± 13.2 mmHg, the mean GCS was 14.8 ± 1.2, and the mean peripheral oxygen saturation was 96.7 ± 5.3% (Table 1). According to the emergency severity index triage system, levels I, II, III, and IV patients comprised 1.78%, 19.68%, 37.25%, and 41.29%, respectively.

**Table 1:**
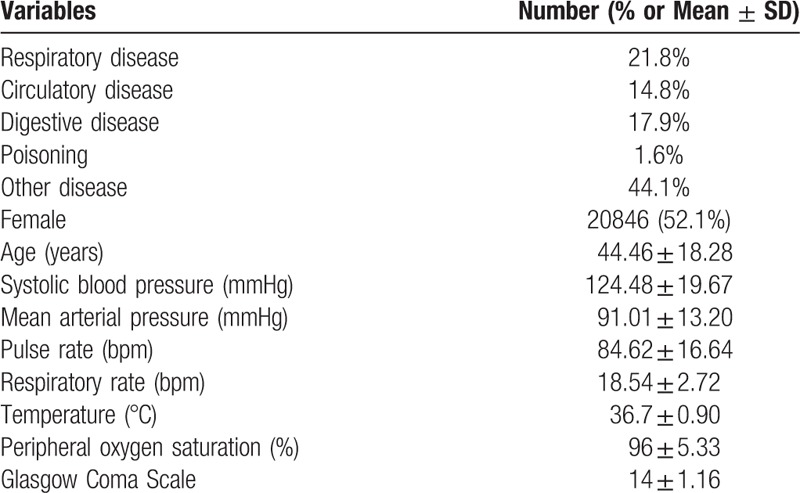
Patients’ characteristics.

### 3.2 Hospital admission

**Figure 1:**
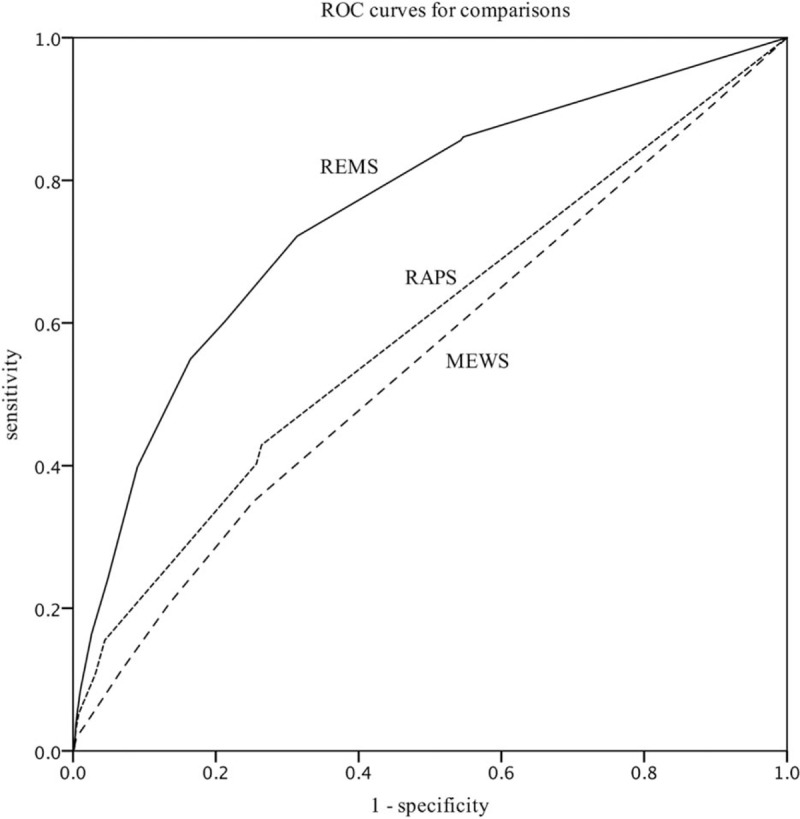
Receiver operating curves for predicting hospital admission according to REMS, RAPS, and MEWS. REMS (AUROC: 0.756, Hosmer-Lemeshow statistic *P* value < .001); MEWS (AUROC: 0.551, Hosmer-Lemeshow statistic *P* value < .001); RAPS (AUROC: 0.592, Hosmer-Lemeshow statistic *P* value < .001). MEWS = modified early warning score, PPV = positive predictive value, RAPS = rapid acute physiology score, REMS = rapid emergency medicine score.

AUROC analysis demonstrated that the predictability of the REMS (AUROC, 0.756; 95% CI, 0.748–0.763) was superior to that of the RAPS (AUROC, 0.59; 95% CI, 0.58–0.60) and the MEWS (AUROS, 0.55; 95% CI, 0.54–0.56) as a predictor of hospital admission **(***P* < .05) (Fig. 1).

### 3.3 In-Hospital mortality

**Figure 2:**
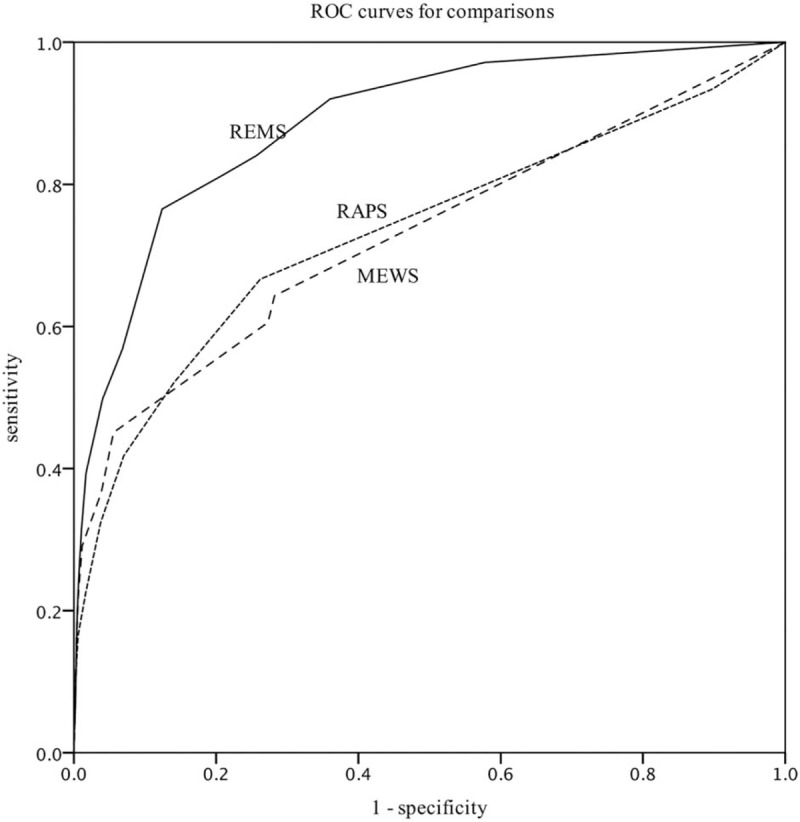
Receiver operating curves for predicting mortality according to REMS, RAPS, and MEWS. REMS (AUROC: 0.886, Hosmer-Lemeshow statistic *P* value < .001); MEWS (AUROC: 0.728, Hosmer Lemeshow statistic *P* value < .001); RAPS (AUROC: 0.733, Hosmer Lemeshow statistic *P* value < .001). MEWS = modified early warning score, RAPS = rapid acute physiology score, REMS = rapid emergency medicine score.

AUROC analysis results showed that the REMS (AUROC, 0.88; 95% CI, 0.86–0.90) was superior to the RAPS (AUROC, 0.72; 95% CI, 0.69–0.77) and the MEWS (AUROC, 0.73; 95% CI, 0.69–0.78) as a predictor of in-hospital mortality **(***P* < .05**)** (Fig. 2).

### 3.4 Length of hospital stay

**Figure 3:**
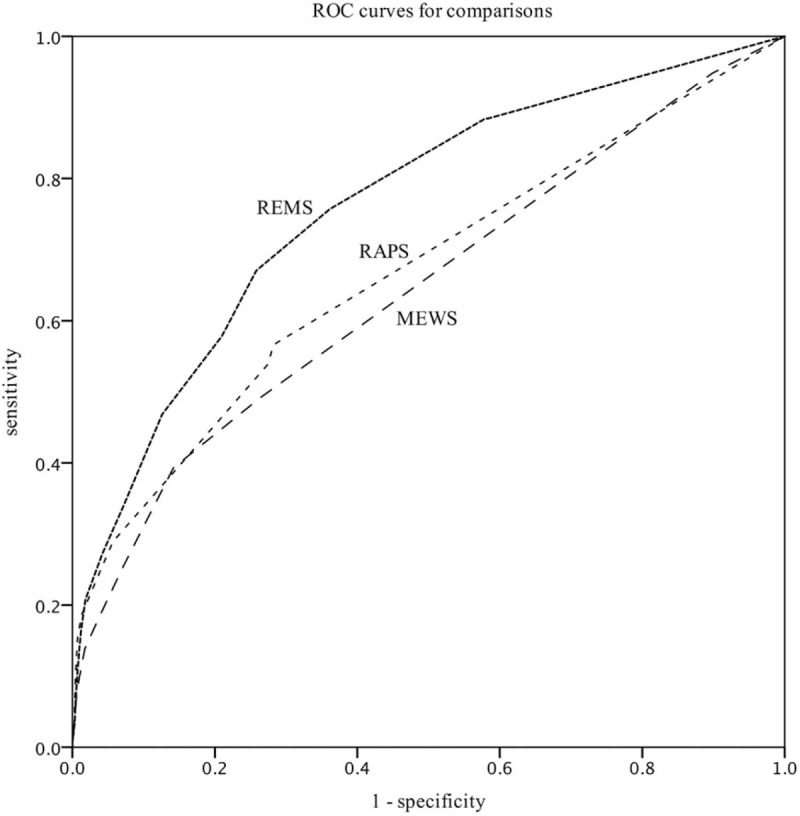
Receiver operating curves for predicting length of hospital stay according to REMS, RAPS, and MEWS. REMS (AUROC: 0.762, Hosmer-Lemeshow statistic *P* value < .001); MEWS (AUROC: 0.645, Hosmer-Lemeshow statistic *P* value < .001); RAPS (AUROC: 0.668, Hosmer-Lemesh-ow statistic *P* value < .001). MEWS = modified early warning score, RAPS = rapid acute physiology score, REMS = rapid emergency medicine score.

The results of AUROC analysis showed that the REMS (AUROC, 0.76; 95% CI, 0.72–0.80) was superior to the RAPS (AUROC, 0.67; 95% CI, 0.62–0.72) and the MEWS (AUROC, 0.65; 95% CI, 0.60–0.70) as a predictor of length of hospital stay **(***P* < .05) (Fig. 3). Moreover, REMS was shown to have the highest sensitivity and specificity among the 3 scoring systems (Table 2).

**Table 2:**
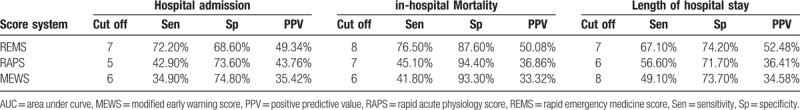
Cut-off, sensitivities, specificities, and positive predictive value of RAPS, MEWS, and REMS.

### 3.5 Sensitivity analysis

To evaluate the predictive power of each of the constituent elements of the RAPS, MEWS, and REMS, we first undertook univariate analysis, using logistic regression to estimate the association between the score for the variable concerned and the outcomes. We then undertook multivariate analysis to determine which individual variables were independent predictors of the outcomes. Multivariate analysis showed that GCS (adjusted odds ratio (aOR), 2.92; 95% CI, 2.68–3.19; *P* < .001), RR (aOR, 2.69; 95% CI, 2.36–3.07; *P* < .001), peripheral oxygen saturation (SpO_2_) (aOR, 2.67; 95% CI, 2.40–2.96; *P* < .001), and MAP (aOR, 2.11; 95% CI, 1.88–2.36; *P* < .001) were independent predictors of mortality. After adjusting for other variables, temperature (aOR, 1.10; 95% CI, 0.88–1.39; *P* = .41) did not predict mortality and appeared to have a weak inverse relationship with mortality. The independent predictors of hospital admission and length of hospital stay are shown in Table 3.

**Table 3:**
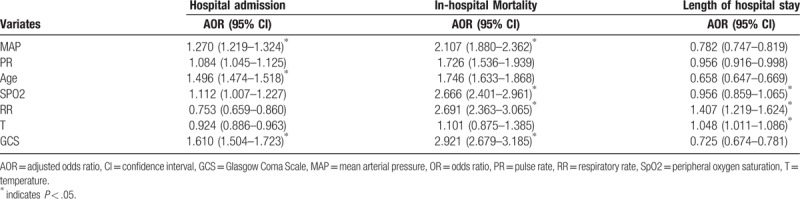
Multivariate analysis and their association with outcomes.

## 4 Discussion

Our study evaluated and confirmed the REMS as a powerful predictor of ED adult patient outcomes, including hospital admission, length of hospital stay, and in-hospital mortality. The REMS significantly predicted in-hospital mortality, suggesting that there is considerable room for improvement in clinical care. This score can also serve to help guide distribution of medical resources as patients with a high REMS score require an urgent and comprehensive medical examination. As this study aimed to identify predictors of outcomes in ED patients, we consider that identifying patients with a good prognosis may help prevent avoidable hospitalizations that achieve little in the way of improving a patient's overall outlook and reduce medical costs.

Numerous studies have identified predictors of patient outcomes. A survey by Lucke et al^[6]^ reported that the demographic and clinical factors of ED visits could be useful in predicting patients who were likely to be admitted to the hospital. A retrospective cohort study conducted by Burch^[7]^ found that the MEWS was less sensitive and specific in predicting mortality in critically ill Asian patients than in non-Asian patients, suggesting that regional variations could influence scoring accuracy and outcomes. APACHE-II, the more sophisticated predictive score, has been reported to have the greatest accuracy, but its variables cannot be easily obtained in the early ED phase. The REMS was developed for non-surgical patients in the ED. The advantages of the REMS are its simplicity and lack of dependence on any laboratory index values. In contrast, Olsson^[8]^ found that the REMS had the same predictive accuracy as the well-established and more complicated APACHE-II score. That study also found that the REMS, as a powerful predictor of in-hospital mortality of ED patients, was superior to the RAPS.^[9]^ Furthermore, Goodacre^[3]^ and Seak^[10]^ showed that the REMS was better than the RAPS in predicting mortality in emergency hospital admissions. Our results are consistent with the above studies.

There are also other methods to predict outcomes. The UK's national early warning score (NEWS) was originally designed to predict death within 24 hours; after which time its discrimination falls, so that a low score cannot be used to justify discharging a patient from the hospital.^[11]^ The quick sequential organ failure assessment (qSOFA) score, which is calculated according to simultaneous vital signs, was associated with hospital admission, in-hospital mortality, ICU admission, and length of hospital stay in ED adult patients, and its predictability has been shown to be even greater than that of MEWS. However, its indices are difficult to obtain initially in an ED setting, and they are not suitable for emergency situations.^[5]^ The mode of arrival and the triage score, which are unique characteristics of ED patients, may predict hospital outcomes for patients with sepsis.^[12]^

With clinical intuition alone, clinical staff in a medical admissions department may still have a good ability to predict those patients at an increased risk of dying. However, a physician's ability to determine a prognosis is sometimes flawed because physicians tend to subjectively overestimate life expectancy.^[13–15]^ Therefore, these intuitive approaches are not suitable for use in the ED.

Once a patient is admitted to hospital, a prognosis can be estimated using an additionally available early laboratory testing index, which appears to carry more predictive power. Marchetti et al^[16]^ showed that the N-terminal pro b-type natriuretic peptide level with age and creatinine clearance on admission were predictors of 30-day mortality in adult patients with acute heart failure. Kong and Kang et al found that a high delta neutrophil index value is a useful marker to predict 28-day mortality in patients with acute pulmonary embolism.^[17,18]^ An increase in troponin is of prognostic value for hospital mortality in critically ill patients.^[19]^ In multiple trauma groups, blood sugar changes in the early hours of admission may help to predict hospital mortality in the ED. Additionally, tachycardia and coagulopathy have been reported to be significantly associated with in-hospital mortality.^[20]^ In the ED, the predictors of outcomes should be readily available in routine clinical practice worldwide; however, all the above indices are difficult to obtain in the initial ED setting and are therefore not suitable for use in the ED. As such, we did not select these variables for our study.

In conclusion, as with previous studies, the REMS offered predictability for adult ED patient outcomes rapidly and continuously. The application of such an approach can help emergency physicians readily identify patients at high risk of mortality, develop their care plan, avoid unnecessarily harmful interventions, and improve the quality of medical care.

## Acknowledgments

All the authors wish to thank Fangjian Yuan (the staff of Zhongnan Hospital Information Center) for the extraction of data, further, we would like to thank Editage (www.editage.com) for English language editing.

## Author contributions

Conceived and designed the study: Yan Zhao. Acquisition of the data: Xiaojun Wei. Analysis and interpretation of the data: Ruining Liu. Drafting of the manuscript: Xiaojun Wei and Haoli Ma.

**Formal analysis:** Xiaojun Wei.

**Project administration:** yan zhao.

**Resources:** Xiaojun Wei.

**Software:** Xiaojun Wei, Ruining Liu.

**Writing – original draft:** Xiaojun Wei.

**Writing – review & editing:** Xiaojun Wei, Haoli Ma.
